# Distribution of ABO and Rh Blood Groups and Allele Frequencies in the Bhutanese Population: A Retrospective Cross‐Sectional Study

**DOI:** 10.1155/bmri/3956641

**Published:** 2026-04-17

**Authors:** Karma Yangchen, Chimi Wangmo, Tshering Choden, Tshering Dorji

**Affiliations:** ^1^ Food Drug and Environment Centre, Royal Centers for Disease Control, Ministry of Health, Royal Government of Bhutan, Thimphu, Bhutan, moh.gov.bt; ^2^ Department of Pathology and Laboratory Medicine, Eastern Regional Referral Hospital, Mongar, Bhutan, lhsc.on.ca; ^3^ Central Regional Referral Hospital, National Medical Services, Ministry of Health, Royal Government of Bhutan, Thimphu, Bhutan, moh.gov.bt; ^4^ Centre for Emerging and Infectious Disease, Royal Centers for Disease Control, Ministry of Health, Royal Government of Bhutan, Thimphu, Bhutan, moh.gov.bt

**Keywords:** ABO grouping, allele frequency, D antigen, phenotype, prevalence, transfusion

## Abstract

**Introduction:**

The prevalence of ABO and Rh blood groups varies among different populations, influencing blood bank management and transfusion safety. This retrospective cross‐sectional study is aimed at analyzing the distribution of ABO and Rh(D) blood groups and their allele frequencies in the Bhutanese population.

**Materials and Methods:**

Retrospective data were collected from five geographically representative hospitals in Bhutan between 2019 and 2023, including three referral hospitals and two district hospitals. The study sample comprised 40,249 individuals, including blood donors, mothers at antenatal clinics, and patients undergoing medical screening during the period January 2019 to December 2023. ABO and Rh blood groups were determined by slide and/or tube agglutination tests in each hospital’s laboratory. Allele frequencies were calculated using Hardy–Weinberg equilibrium principles.

**Results:**

In this retrospective cross‐sectional study of 40,249 individuals from five hospitals across Bhutan, the most common ABO blood group observed was O (*n* = 13,993; frequency = 0.347), followed by A (*n* = 13,015; 0.323), B (*n* = 9742; 0.242), and AB (*n* = 3499; 0.086). Rh(D)‐positive individuals accounted for the vast majority (*n* = 40,105; 0.9964), while Rh(D) negative was rare (*n* = 144; 0.0035). The allele frequencies were 0.229 for *I* ^A^, 0.178 for *I* ^B^, and 0.589 for *I* ^O^ in the ABO system and 0.940 for *I* ^D^ and 0.059 for *I* ^d^ in the Rh(D) system.

**Conclusions:**

This study highlights the predominance of blood group O and Rh(D) positivity in the Bhutanese population. The allele distribution shows a higher frequency of the *I* ^O^ and *I* ^D^ alleles, indicating genetic homogeneity in blood group distribution. These findings provide essential baseline data for transfusion medicine, population genetics, and future public health planning.

## 1. Introduction

The ABO blood group system is one of the most clinically significant human blood groups, classifying human populations into four phenotypes (A, B, AB, and O) based on the presence of carbohydrate antigens on erythrocytes and other tissues. ABO antigens and corresponding naturally occurring antibodies are essential for transfusion medicine, antenatal care, and population genetics [1, 2]. The ABO system is determined by a single gene locus on Chromosome 9 (9q34) with three primary alleles *I* ^A^, *I* ^B^, and *I* ^O^ that code for A, B, and O antigens, respectively. It follows a Mendelian pattern of inheritance, meaning it is passed from parents to children according to basic genetic rules discovered by Gregor Mendel. The alleles *I* ^A^ and *I* ^B^ are codominant, while both are dominant over *I* ^O^, which results in blood group O [3, 4].

The Rh blood group system is the second most clinically important blood group system after ABO and is characterized by extensive genetic and antigenic complexity. The most significant antigen of the Rh system is the D antigen, which determines Rh(D) positivity or negativity and is a major cause of alloimmunization in transfusion medicine and pregnancy [5]. Genetically, Rh(D)‐positive individuals carry at least one D allele (DD or Dd), whereas Rh(D)‐negative individuals are homozygous for the absence of the D allele (dd) [6]. Hemolytic disease of the newborn (HDN) due to Rh incompatibility is an isoimmune hemolytic disorder in which maternal anti‐D antibodies cross the placenta in subsequent pregnancies and attack fetal Rh‐positive red blood cells. This immune response results in hemolysis, causing fetal anemia, jaundice, and, in severe cases, hydrops fetalis or intrauterine death [7].

The distribution of ABO and Rh(D) blood groups shows considerable variation across populations worldwide and among different regions within a country. Population‐specific data on ABO and Rh(D) phenotypes and allele frequencies are essential for efficient blood bank inventory management [8]. In addition to their clinical relevance in transfusion medicine, ABO and Rh blood group systems serve as informative genetic markers in population studies, migration research, and medicolegal contexts, particularly in cases of disputed parentage. ABO and Rh(D) blood group distributions have been extensively reported in neighboring South Asian populations, such as India [9]. However, in Bhutan, comprehensive nationwide data remain limited, with previous studies only focused on donor populations in a single region [10]. Therefore, this study is aimed at determining the nationwide distribution of ABO and Rh(D) blood groups in Bhutan and at estimating their allele frequencies using a multicenter dataset representing different geographic regions. The findings will provide essential baseline data for transfusion medicine, antenatal management, and population genetic studies in Bhutan.

## 2. Materials and Methods

### 2.1. Study Design and Setting

**Figure 1:**
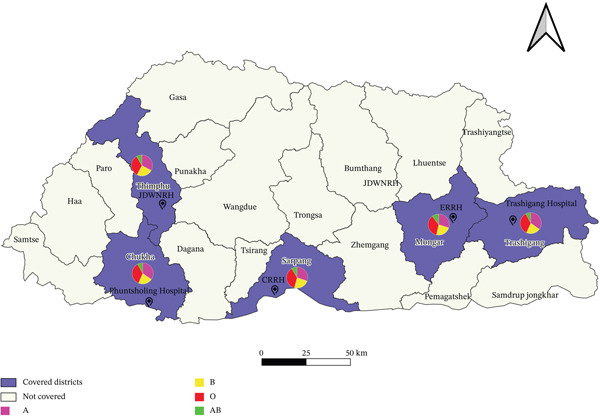
Geographic distribution of selected study sites with ABO blood group proportions.

This retrospective cross‐sectional study analyzed data collected between January 2019 and December 2023 from five major hospitals in Bhutan. The hospitals were selected to represent the country’s major geographic regions and population diversity. Jigme Dorji Wangchuck National Referral Hospital (JDWNRH) represented the western region, Central Regional Referral Hospital (CRRH) represented the central region, Phuentsholing Hospital represented the southern region, and Eastern Regional Referral Hospital (ERRH) along with Trashigang Hospital represented the eastern region. The selected facilities included both regional referral and district hospitals, which serve as tertiary healthcare centers for their surrounding catchment areas. This geographic distribution ensured that the study population included individuals from diverse population densities, ethnic groups, and demographic backgrounds, reflecting the overall national population (Figure 1).

### 2.2. Sample Selection and Data Sources

The study included all individuals who underwent blood grouping during the study period. This comprised (1) voluntary and replacement blood donors, (2) pregnant women attending antenatal clinics, and (3) patients undergoing routine medical screening.

Blood transfusion service data were obtained through the National Blood Safety Program database, while antenatal and screening records were retrieved from hospital archives and registers. The hospital archives contained a larger number of total records; however, only records that met the predefined inclusion criteria were entered into the Excel database for analysis. Records that did not meet the predefined criteria were present in the hospital records but were excluded from the dataset. Therefore, the final dataset comprised 40,249 individuals, including 24,997 blood donors, 15,132 pregnant women attending antenatal clinics, and 120 patients undergoing routine medical screening. All included records fulfilled the inclusion criteria, and no additional filtering or removal of records was required during the data processing stage.

### 2.3. Inclusion and Exclusion Criteria

• Inclusion criteria: All records containing complete information on both ABO and Rh(D) blood group status.
• Exclusion criteria: Records with missing or incomplete information, unclear serological results, and duplicate records from the same individual. Only records meeting the predefined inclusion criteria were entered into the dataset for analysis.

### 2.4. Allele Frequency Estimation

Allele frequencies for the ABO and Rh(D) blood group systems were calculated using the Hardy–Weinberg equilibrium (HWE). For the ABO system, the expected genotype distribution was expressed as follows:

p2+2221pr+r2+q2+qr+pq=,

where *p*, *q*, and *r* represent the allele frequencies of *I* ^A^, *I* ^B^, and *I* ^O^, respectively. Allele frequencies were derived from the observed phenotype frequencies using the following equations:

p=11−B+O,q=−A+O,r=O.

For the Rh(D) system, allele frequencies were estimated as follows:

E2+21Ee+e2=,

where *E* and *e* represent the frequencies of the Rh(D)‐positive (D) and Rh(D)‐negative (d) alleles. The *d* allele frequency was calculated as follows:

e=dd,E=1−e.

This approach has been widely applied in population studies of ABO and Rh(D) blood groups [6, 11, 12].

### 2.5. Statistical Analysis

The ABO and Rh(D) phenotype data were tabulated and analyzed using Microsoft Excel. We computed descriptive statistics (frequency and percentage) for each blood group. Allele frequencies were then calculated using the HWE formulas given above. All statistical computations (e.g., frequency counts, percentages, and allele frequency calculations) were performed in Excel, and results were checked for consistency with Hardy–Weinberg expectations. Differences in ABO and Rh blood group distributions across hospitals were calculated in R using the chi‐square test of independence for ABO groups and Fisher’s exact test for Rh groups. Effect sizes for ABO distributions were calculated using Cramér’s *V*, with statistical significance defined as *p* < 0.05.

## 3. Results

From January 2019 to December 2023, a total of 40,249 individuals visited five hospitals for blood donation, antenatal checkups, and medical screening. Among these visits, 21,102 (52.5%) were male, while 19,147 (47.5%) were female (Table 1).

**Table 1:** Distribution of study participants by sex across hospitals (2019–2023).

Hospital	Male (*n*, %)	Female (*n*, %)	Total (*n*)
Phuentsholing Hospital	2361 (47.5)	2611 (52.5)	4972
Trashigang Hospital	1232 (74.3)	425 (25.7)	1657
Jigme Dorji Wangchuck National Referral Hospital	9976 (43.3)	13,040 (56.7)	23,016
Central Regional Referral Hospital	3814 (67.0)	1883 (33.0)	5697
Eastern Regional Referral Hospital	3719 (75.7)	1188 (24.3)	4907
Total	21,102 (52.5)	19,147 (47.5)	40,249

*Note:* Percentages are calculated row‐wise, based on the total number of participants enrolled from each hospital between January 2019 and December 2023.

Blood group O was the most prevalent phenotype at all study sites, with frequencies ranging from 0.337 at JDWNRH to 0.370 at CRRH. Blood group A was the second most common phenotype in Phuentsholing Hospital (0.346), Trashigang Hospital (0.348), and JDWNRH (0.327), whereas blood group B showed a relatively higher frequency at JDWNRH (0.249) compared with other hospitals. Blood group AB was consistently the least prevalent phenotype across all hospitals, although its frequency was comparatively higher at ERRH (0.103). In contrast, the distribution of the Rh(D) blood group was highly uniform across the hospitals. Rh(D)‐positive individuals constituted more than 99.5% of the population at each site, with frequencies ranging from 0.9956 at Phuentsholing Hospital to 0.9994 at Trashigang Hospital. In this study, the phenotypic distribution of ABO blood groups differed significantly across the five hospitals (*χ* ^2^ = 87.21, df = 12, *p* < 0.001). The proportion of Rh(D)‐negative individuals remained low across all hospitals and did not differ significantly (Fisher’s exact test, *p* = 0.198) (Table 2).

**Table 2:** Distribution and phenotype frequencies of ABO and Rh blood groups across hospitals.

Hospital	A, *n* (*f*)	B, *n* (*f*)	AB,*n* (*f*)	O,*n* (*f*)	Rh(D)+,*n* (*f*)	Rh(D)−,*n* (*f*)	Total
Phuentsholing Hospital	1721 (0.346)	1095 (0.220)	401 (0.080)	1755 (0.353)	4950 (0.9956)	22 (0.0044)	4972
Trashigang Hospital	577 (0.348)	363 (0.219)	131 (0.079)	586 (0.353)	1656 (0.9994)	1 (0.0006)	1657
JDWNRH	7531 (0.327)	5748 (0.249)	1963 (0.085)	7774 (0.337)	22,934 (0.9964)	82 (0.0036)	23,016
CRRH	1722 (0.302)	1373 (0.241)	494 (0.086)	2108 (0.370)	5676 (0.9963)	21 (0.0037)	5697
ERRH	1464 (0.298)	1163 (0.237)	510 (0.103)	1770 (0.360)	4889 (0.9963)	18 (0.0037)	4907
Total	13,015 (0.323)	9742 (0.242)	3499 (0.086)	13,993 (0.347)	40,105 (0.9964)	144 (0.0036)	40,249

*Note:* *n*, number of individuals with the corresponding blood group; *f*, phenotype frequency.

In this study, the overall prevalence of ABO blood groups among 40,249 individuals was as follows: blood group O was the most common with a prevalence of 34.7%, followed by group A at 32.3%, group B at 24.2%, and group AB at 8.6%. Regarding the Rh(D) blood group system, Rh(D)‐positive individuals constituted the vast majority with a prevalence of 99.64%, while Rh(D)‐negative individuals accounted for only 0.36% of the study population (Table 3).

**Table 3:** Prevalence rate of ABO and Rh blood groups.

Blood group system	Phenotype	Total (*n*)	Phenotype frequency	Prevalence (%)
ABO	A	13,015	0.323	32.3
	B	9742	0.242	24.2
	AB	3499	0.086	8.6
	O	13,993	0.347	34.7
Rh(D)	Rh(D) positive	40,105	0.9964	99.64
	Rh(D) negative	144	0.0035	0.36
Total		40,249	1.00	100

The allele frequencies of ABO and Rh(D) blood group genes calculated based on HWE were 0.229 for *I* ^A^, 0.178 for *I* ^B^, 0.589 for *I* ^O^, 0.940 for *I* ^D^, and 0.059 for *I* ^d^ (Table 4).

**Table 4:** Distribution of allele frequency of ABO and Rh blood groups.

Blood group system	Blood group allele	Allele frequency
ABO	*I* ^A^	0.229
	*I* ^B^	0.178
	*I* ^O^	0.589
Rh(D)	*I* ^D^	0.940
	*I* ^d^	0.059

## 4. Discussion

This multicenter 5‐year study among 40,249 participants from five Bhutanese hospitals is the first study providing a detailed analysis of the prevalence of ABO and Rh(D) blood group distributions in the country. Our findings show that blood group O (34.7%) is the most common, followed by A (32.3%), and B (24.2%) and AB (8.6%) show relatively lower prevalence rates. Rh(D) positivity is nearly universal at 99.64%, with Rh(D) negativity observed in only 0.36% of individuals. Our observed order of phenotypes (O > A > B > AB) confirms and agrees with the findings reported previously among blood donors at CRRH [10]. Nevertheless, our more comprehensive sampling shows a modest reduction in O and a relative increase in A. This may be due to the expanded geographic coverage and diversified sampling strategy of the present study. Although Pearson’s chi‐square test indicated a statistically significant difference in ABO blood group distribution across hospitals (*p* < 0.001), the proportional differences were small and the overall distribution patterns were highly similar. The large and uneven sample sizes across hospitals likely increased the statistical power to detect even minor deviations. This is further supported by a very small effect size (Cramér’s *V* ~ 0.03), suggesting that while these regional variations exist, they carry limited practical or clinical relevance. The findings from our work provide an essential national baseline with immediate practical importance for transfusion services and obstetric care.

Compared with neighboring South Asian groups, Bhutan’s ABO and Rh profile shows both similarities and significant differences. Broad analyses within India show nearly equal O (34.6%) and B (34.1%) frequencies, with A reduced (23.2%), AB least (8.2%), and Rh(D)‐negative prevalence of 5.8% [3]. Nevertheless, northeastern Indian states show a similar pattern to Bhutan with O (36.1%), A (30.7%), B (24.7%), and AB (8.6%) [13]. Bangladesh and Pakistan also report B dominance over A, with Rh(D)‐negative percentage of 4%–10% [14–18]. In contrast, a study from Nepal highlighted A (34.0%) and O (32.6%) as codominant, followed by B (27.3%) and AB (6.1%), with Rh(D)‐negative prevalence of 3.1% [19]. This pattern closely resembles findings in our current study, where A exceeds B and O remains modal, distinguishing both Himalayan populations from the Indo‐Aryan plains. Nationwide surveys conducted in China similarly report O > A > B > AB, though Tibetan subgroups exhibit elevated B frequencies, highlighting fine‐scale heterogeneity across the Himalayan arc [20]. Collectively, these comparisons suggest that Bhutan occupies an intermediate position within South and East Asia, where O remains the modal phenotype, but A is relatively prominent compared with several neighboring populations. Collectively, these comparisons position Bhutan alongside Nepal and other highland groups in a broader Himalayan cline, where A and O together predominate and B frequencies are reduced compared to South Asian lowland populations.

The relatively rare frequency of *I* ^B^ compared with many South Asian populations is consistent with demographic histories shaped by high‐altitude East Asian ancestry and long‐term genetic continuity along the Himalayan arc. Archaeogenetic analyses have documented such continuity and revealed a genetic cline that plausibly explains Bhutan’s deviation from Indo‐Aryan profiles [21]. While serological data alone are insufficient for explaining the underlying mechanisms, integration with genomic analyses could elucidate the balance of genetic drift, admixture, and selection shaping ABO distributions in high‐altitude populations.

The nearly universal Rh(D) positivity has direct and practical implications, and blood banks must plan accordingly. Given the scarcity of Rh(D)‐negative individuals, O‐negative blood should be considered a strategic resource. Establishing a national donor registry for rare blood types and integrating interhospital sharing mechanisms for rare phenotypes are high priorities. Despite having an extremely low frequency of Rh(D) negative, antenatal screening and appropriate prophylaxis remain crucial, as isolated cases of alloimmunization can have severe fetal and neonatal consequences. Molecular characterization of serologically Rh(D)‐negative pregnancies would allow more precise risk stratification for HDN.

There are limitations to our study. Our sampling frame is hospital‐based (blood donors, antenatal visits, and screening attendees) and may not fully represent the general population, and community surveys would complement these findings. Serological testing does not identify all molecular variants (e.g., weak D, partial D, DEL, or rare subtypes of the ABO subgroup), and genotype follow‐up is necessary for the resolution of clinically important exceptions. Lastly, although regional and archaeogenetic surveys put ancestral interpretations into perspective, we need direct genome‐wide data from properly stratified cohorts of Bhutanese populations for verification of the demographic mechanisms proposed here.

In conclusion, Bhutan’s ABO and Rh profile is characterized by O predominance, a relatively high frequency of A, and a very low prevalence of Rh(D) negative. These findings constitute an essential epidemiologic resource that could be used for transfusion policy, antenatal management, and population genetic studies in Bhutan.

## Author Contributions

**Karma Yangchen:** conceptualization, methodology, software, validation, formal analysis, data curation, writing – original draft, writing – review and editing, visualization, supervision. **Chimi Wangmo:** conceptualization, methodology, validation, data curation, writing – original draft, writing – review and editing. **Tshering Choden:** conceptualization, methodology, validation, data curation, writing – original draft, writing – review and editing. **Tshering Dorji:** conceptualization, methodology, software, validation, formal analysis, data curation, writing – original draft, writing – review and editing, visualization.

## Funding

No funding was received for this manuscript.

## Disclosure

The authors alone are responsible for the views expressed in this article, and they do not necessarily represent the views, decisions, or policies of the institutions with which they are affiliated.

## Ethics Statement

Ethical clearance for this study was obtained from the Research Ethics Board of Health (REBH), Ministry of Health, Bhutan (Approval No. 2026.42NW). Permission to access hospital records and blood bank data was granted by the National Medical Services and the administrations of the participating hospitals. All data were fully anonymized before analysis, and no personal identifiers were recorded. As a retrospective study, the requirement for individual informed consent was waived by the REBH.

## Conflicts of Interest

The authors declare no conflicts of interest.

## Data Availability

All data required to support the conclusions of this article are presented in the form of direct quotes within the manuscript. Raw data containing personally identifiable and sensitive information cannot be made publicly available. Reasonable requests for the data may be considered by the corresponding author upon formal request but are subject to ethical and privacy regulations.
